# Postponement of the opacification of lentoid bodies derived from human induced pluripotent stem cells after lanosterol treatment—the first use of the lens aging model *in vitro* in cataract drug screening

**DOI:** 10.3389/fphar.2022.959978

**Published:** 2022-08-19

**Authors:** Lifang Zhang, Zhenwei Qin, Danni Lyu, Bing Lu, Zhijian Chen, Qiuli Fu, Ke Yao

**Affiliations:** ^1^ Eye Center of the 2nd Affiliated Hospital, School of Medicine, Zhejiang University, Zhejiang Provincial Key Lab of Ophthalmology, Hangzhou, China; ^2^ Department of Ophthalmology, The Affiliated People’s Hospital of Ningbo University, Ningbo, China; ^3^ Department of Environmental and Occupational Health, Zhejiang Provincial Center for Disease Control and Prevention, Hangzhou, China

**Keywords:** age-related cataracts, lentoid body, lanosterol, crystallin aggregation, drug screening

## Abstract

**Purpose:** Our previous study observed that human induced pluripotent stem cell (HiPSC)-derived lentoid bodies (LBs) became cloudy with extended culture time, partially mimicking the progress of human age-related cataracts (ARCs) in a dish. In the present study, lanosterol, a potential anticataract drug, was used to further verify the value of this model in drug screening for cataract treatment.

**Methods:** Mature LBs on day 25, which were differentiated from HiPSCs using the “fried egg” method, were continually cultured and treated with either dimethyl sulfoxide (control) or lanosterol. The LBs’ shape and opacity alterations were examined using light microscopy and mean gray value evaluation. The soluble and insoluble proteins were examined through SDS-PAGE gel electrophoresis combined with Coomassie blue staining. The protein aggregations were examined with immunofluorescence.

**Results:** The mature LBs became cloudy with an extended culture time, and the opacification of the LBs was partially prevented by lanosterol treatment. There was less increase in insoluble proteins in the lanosterol-treated LBs than in the control group. There were also fewer cells containing aggregated protein (αA‐crystallin and αB‐crystallin) puncta in the lanosterol-treated LBs than in the control LBs.

**Conclusion:** It was found that the opacification of LBs could be delayed by lanosterol treatment, which could be achieved by reducing protein aggregation, suggesting a promising HiPSC-derived drug-screening model for Age-related cataract.

## Introduction

The human lens is a uniquely transparent, biconvex, avascular structure responsible for focusing an image onto the retina. Progressive loss of lens transparency is accompanied by a significant fall in the rate and amplitude of accommodation with the aging process. The latter is the basis of presbyopia while the former can cause light scatter and visual impairment, and eventually leads to cataracts. (Michael and Bron, 2011).

Age-related cataract (ARC) is one of the leading causes of blindness worldwide, and its prevalence increases with age, reaching 92.6% at 80 years and older. (Liu et al., 2017). ARCs can be divided into three types according to the location of opacification within the lens: nuclear, cortical, and posterior subcapsular cataracts. Cataract severity can be determined by grading cortical (C), nuclear-color or nuclear-opalescence (N), and posterior subcapsular (P) cataracts according to the Lens Opacities Classification System II. (Chylack et al., 1989). Most cataracts develop slowly and do not disturb the eyesight early on, but over time, they will progress, eventually impairing the patients’ vision and interfering with their daily activities.

Surgery remains the only effective treatment for cataract patients with sufficient-severity vision loss. With surgical removal of the cataractous lens and replacement with an intraocular lens, most patients could regain good vision. (Chen et al., 2021). However, given the lack of access to surgical care in many parts of the world, a large number of cataract patients cannot obtain effective treatment, which makes cataract a major cause of sight loss. In addition, surgery may be followed by complications, such as lens capsular tear, posterior capsular opacification, and intraocular infections. It also imposes a financial burden on patients and the healthcare system. The World Health Organization has reported that United States $6.9 billion is needed to treat the existing unaddressed cases of cataract globally. (WHO, 2019). It has been estimated that a one-decade delay in the onset of ARC will reduce the surgical and associated costs by 50%. (WHO, 2019). Therefore, the need to produce effective therapeutic treatments, such as drug therapy, is as important as ever, especially for mild cataracts. To date, no conclusive pharmacological treatment has been developed for the effective treatment of human cataracts.

The search for anticataract drugs has continued for decades. The induction of effective cataract models is one of the key steps in anticataract drug screening. At present, the commonly used cataract models include human lens cell lines, *in vitro* lens organ models, and *in vivo* animal models, such as rats, mice, rabbits, and dogs. Some drugs, such as aspirin and aspirin-like drugs, protein stabilizers/protectors, opioid growth factor, and exogenous antioxidants, have shown promising results with these cataract models. Two recent studies have also found that lanosterol and its derivatives have the ability to depolymerize aggregated proteins and thereby restore partial vision in animals. (Makley et al., 2015; Zhao et al., 2015). However, the clinical application of the two components was restricted due to their physical and chemical properties. Thus, more effective lanosterol derivatives need to be sought as they are among the most promising cataract drug treatment modalities. Toward this end, an appropriate and effective drug-screening system including cataract disease models not only containing human origins but also mimicking the disease progress *in vivo* is needed. An HiPSC-derived cataract disease model may qualify as such system.

Because of the unavailability of human tissue, scientists attempt to regenerate human tissue from pluripotent stem cells (PSCs) such as those from the gut, kidney, brain, and retina for pathological mechanism investigation and drug testing of various diseases. (Bellin et al., 2012; Lancaster and Knoblich, 2014). For example, gut organoids are already being used to examine infectious diseases, tumor biology, and genetic conditions, and *in vitro* human liver models are being used to screen compounds and to be part of the drug discovery process. Regeneration of the crystalline lens is one of the hot research topics in this area. Based on the previous study, a “three‐step” system for differentiating lentoid bodies (LBs) from human embryonic stem cells (ESCs), (Yang et al., 2010), we have established and improved the lens regeneration method, and during our study, LBs derived from HiPSCs showed three-dimensional transparent morphologies and basic optic characteristics. (Fu et al., 2017). Moreover, the LBs not only expressed lens-specific markers but also developed complete capsules and epithelial cells. (Fu et al., 2017). A continued study showed that the transparent mature LBs became cloudy with an extended incubation period accompanied by protein aggregation, a process that could be accelerated by hydrogen peroxide, partially showing the progress of human lens aging or ARCs in another term. (Qin et al., 2019). Very recently, regenerated lenses showing obvious opacification closely resembling that seen in patients’ cataracts in terms of opacification severity and disease course accordingly were developed using HiPSCs derived from congenital-cataract patients. (Lyu et al., 2021). What’s more, as the first try in drug screening based on the HiPSC-derived LBs, the opacification of patients’specific regenerated lenses was attenuated by lanosterol treatment, (Lyu et al., 2021), indicating the potential use of this model in anticataract drug screening.

In the present study, we aim to verify the possibility of opacitication LB model with extending incubation in the potential application of anticataract drug screening based on the effects of lanosterol treatment, and to analyze whether it is involved change of protein aggregation.

## Methods and materials

### Cell culture and lentoid body differentiation

Urinary cell‐derived iPSCs (UiPSCs) were generated and characterized in our previous study. (Fu et al., 2017). UiPSCs were seeded in Matrigel‐coated (B&D) dishes, cultured with an mTesR medium (STEMCELL), and passaged with 0.5 mM ethylene diamine tetraacetic acid in phosphate buffer saline (PBS) every 4 days. (Beers et al., 2012). The “fried egg” method for the differentiation of LBs from UiPSCs/ESCs was described in detail in our previous article. (Fu et al., 2017). In brief, cells were triggered with 100 ng/ml BMP‐inhibitor Noggin for 6 days until the wanted cell clusters were mechanically isolated and reseeded in new dishes. A mixture of BMP4 (20 ng/ml), BMP7 (20 ng/ml), and bFGF (100 ng/ml) was then added to the medium for 9 days. A typical “fried egg”‐like structure appeared at around day 11 (D11) of differentiation. Finally, BMP4 and BMP7 were replaced with Wnt3a (20 ng/ml) to accomplish the final differentiation. Mature LBs were obtained at around day 25 (D25).

### Drug treatment

Lanosterol compound (4 mM; Sigma-Aldrich) was dissolved in dimethyl sulfoxide (DMSO; Sigma-Aldrich). Beginning on D25, 4 μM lanosterol was applied to the mature LBs every day. Mature LBs treated with 0.1% DMSO served as controls.

### Morphological observation and transparent evaluation of lentoid bodies

The LBs’ morphologies were examined daily, and representative images were taken using light microscopy, which was used to evaluate the LBs’ degrees of transparency, as mentioned in our previous article. (Qin et al., 2019).

The selection criteria for LBs. During LBs induction, we were unable to standardize the size, shape and volume of LBs. Therefore, we can only select enough LBs and use the “gray value measurement” to conduct quantitative transformation and statistical analysis of their transparency. Our criteria for selecting LBs is to have clear borders and transparency at D25.

Gray value measurement. We observed the photos of LBs and found that the more transparency of LB, the whiter of this region, so we use ImageJ software to transfer the photos into grayscale images which only has two-dimensional of colors: black and white, and then calculated the grey value of each pixel on the chosen region. We use the mean grey value to represent the transparency of LB and then realized the quantization of transparency. The following are the detection steps of “gray value measurement”, first we took photos of LBs, second, we transferred them into grayscale images, third we marked the calculation region and got the mean gray value of pixel on this region. The gray value of white was two hundred and fifty-six, and the black was zero. The decreasing of LB in gray value indicated the decrease of its transparency. Then, the mean gray values of the LBs in each condition were compared. In addition, the decreased rate of the transparent degree was calculated as the ΔGray value: the gray value of the day: Day (n), minus the gray value of 3 days later: Day (n + 3), divided by the number of days: 3, that is the decreased rate of LBs transparent over time, where

ΔGray value=(gray value Day (n)-gray value Day (n+3))/3

### Soluble and insoluble protein concentration determination

LBs in different conditions and at different time points were lysed and extracted using the Tissue or Cell Total Protein Extraction Kit (Sangon Biotech, Shanghai, China). In brief, after being washed with cold PBS, LBs were lysed in 100 μl lysis buffer with phenylmethylsulfonyl fluoride, phosphatase inhibitor, and protease inhibitor, and were ground with a grinding stick to form a homogenate. Then, the homogenate was vortexed three times, every 5 min. A 40 μl mixture was taken as the total protein, and the remaining part of the mixture was centrifuged at 12,000 g for 20 min at 4°C. The supernatant was separated as soluble protein, and the precipitation to which the same volume of lysate as that in the supernatant was added was insoluble protein.

SDS-PAGE gel electrophoresis combined with Coomassie blue staining was used to quantify the aggregation of Crystallins. After the homogenate was centrifuged to remove the soluble supernatant, the same volume of solution as the supernatant was added and resuspended (insoluble fraction), the ratio of soluble to insoluble protein in each sample was not affected by the protein concentration. Crystallins are the most important structural proteins in lens cells and all crystallin are around 20 kd region. They are water-soluble proteins when perfectly maintaining their dimeric structure, however, when they aggregate, their water solubility decreases and become water-insoluble proteins. Therefore, water-insoluble proteins around 20 kD region can represent aggregated crystallins. The ratio of soluble to insoluble protein in each sample was not affected by the protein concentration, so instead of calculating the concentration of total soluble and insoluble protein, we calculated the soluble and insoluble protein ratios of crystallins (20 kd region) on SDS PAGE gel electrophoresis. In brief, the same volume of all the proteins were loaded into an SDS-PAGE gel and dyed with Coomassie Blue Super-Fast Staining Solution (Beyotime, Shanghai, China) for 30 min. After being decolorized overnight, the bands of proteins were detected using a Bio-Rad Gel Doc XR Imaging System (Bio-Rad). Finally, quantitative analysis of bands around 20 kD region was performed with the ImageJ software.

### Immunofluorescence examination

It is well verified by other researches that crystallin aggregation appear as aggregated fluorescent spots on immunofluorescence staining. Therefore, we quantitatively assessed crystallin aggregation by counting the number of aggregated fluorescent spots on immunofluorescence images. (Zhao et al., 2015; Qin et al., 2019; Lyu et al., 2021). The LBs in the different conditions were fixed with paraformaldehyde (4% in PBS; Sigma-Aldrich), permeabilized with 0.3% Triton X‐100 (Sigma‐Aldrich), and incubated overnight with rabbit anti-αA-crystallin antibody (1/100; Enzo) or rabbit anti-αB-crystallin antibody (1/100; Enzo). Subsequently, the LBs were incubated with Alexa Fluor‐488‐labeled secondary antibody (1/1,000; Invitrogen). Finally, the nuclei were labeled with 4′,6‐diamidino‐2‐phenylindole (0.5 mg/ml; Sigma‐Aldrich). The images were captured using a Leica TCS SP8 confocal microscope (Leica). The percentage of cells with crystallin aggregates was calculated from 10 randomly selected viewing fields.

As for frozen section staining, LBs in the different conditions were embedded in optimal cutting temperature compound (SAKURA Tissue-Tek) and sectioned into 7 mm parts using a Leica CM1950 freezing slicer (Leica). Then, the αA- and αB-crystallin aggregates analysis in slices was performed as was described above.

### Statistical analysis

One-way analysis of variance was performed on all the experiments using the SPSS software (Version 17.0, SPSS). This was followed by least-significant-difference post hoc multiple comparisons when more than two groups required comparison. Statistical significance was defined as *p < .05, **p < .01, and ***p < .001.

## Results

### Lanosterol partially prevented the opacification of the lentoid bodies with extended culture time

**FIGURE 1:**
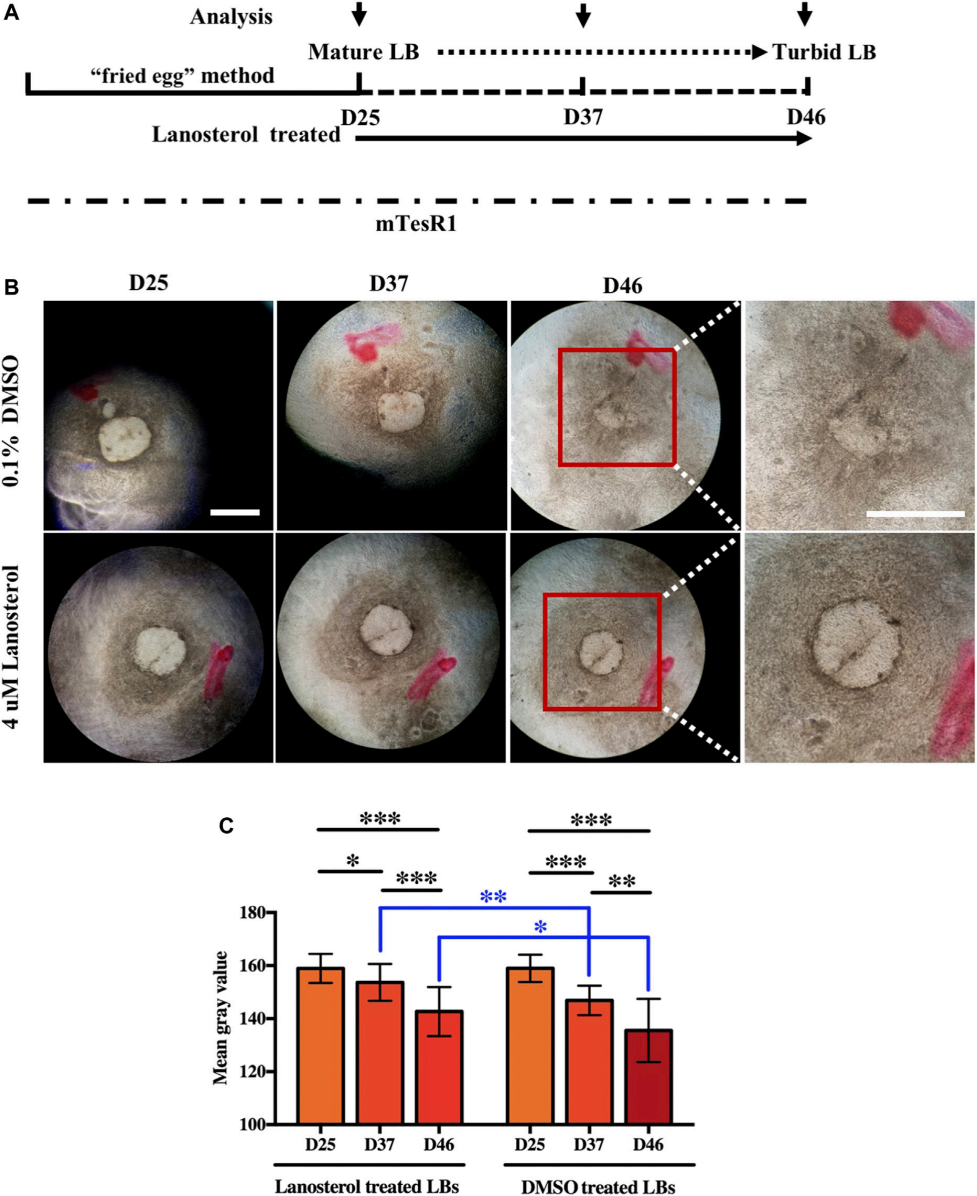
Lanosterol alleviates the opacity of LBs with extended culture time. **(A)** The schematic diagram of LB generation and lanosterol treatment. **(B)** Clouding in the LBs with longer incubation was alleviated after lanosterol treatment. Representative pictures of LBs on D25, D37, and D46 were shown. **(C)** The opacity degrees of LBs were evaluated by mean gray value using the ImageJ software. At least 15 LBs in each group from three independent experiments were examined. Bars represent mean ± SD. *p < .05, **p < .01, ***p < .001. Scale bar:1 mm. DMSO: dimethyl sulfoxide.

LBs with transparent morphologies were differentiated from UiPSCs using the “fried egg” method. Beginning on D25, mature LBs were continuously cultured under DMSO (control) or lanosterol treatment and analyzed under light microscopy at certain time points (Figure 1A). The light microscopy analysis showed that the control LBs gradually lost their transparent morphologies and clear boundaries. The lanosterol-treated LBs were more transparent than the control LBs with a longer incubation period. Representative pictures of the LBs on D25, D37, and D46 are shown in Figure 1B. Quantification analysis revealed that the mean gray value of the LB areas in both groups decreased with time, with that of the control group decreasing more than that of the lanosterol-treated group, demonstrating that the LBs’ opacity became severe with time and that natural opacity could be partially alleviated by drug treatment (Figure 1C).

### Development of lanosterol-treated lentoid bodies

**FIGURE 2:**
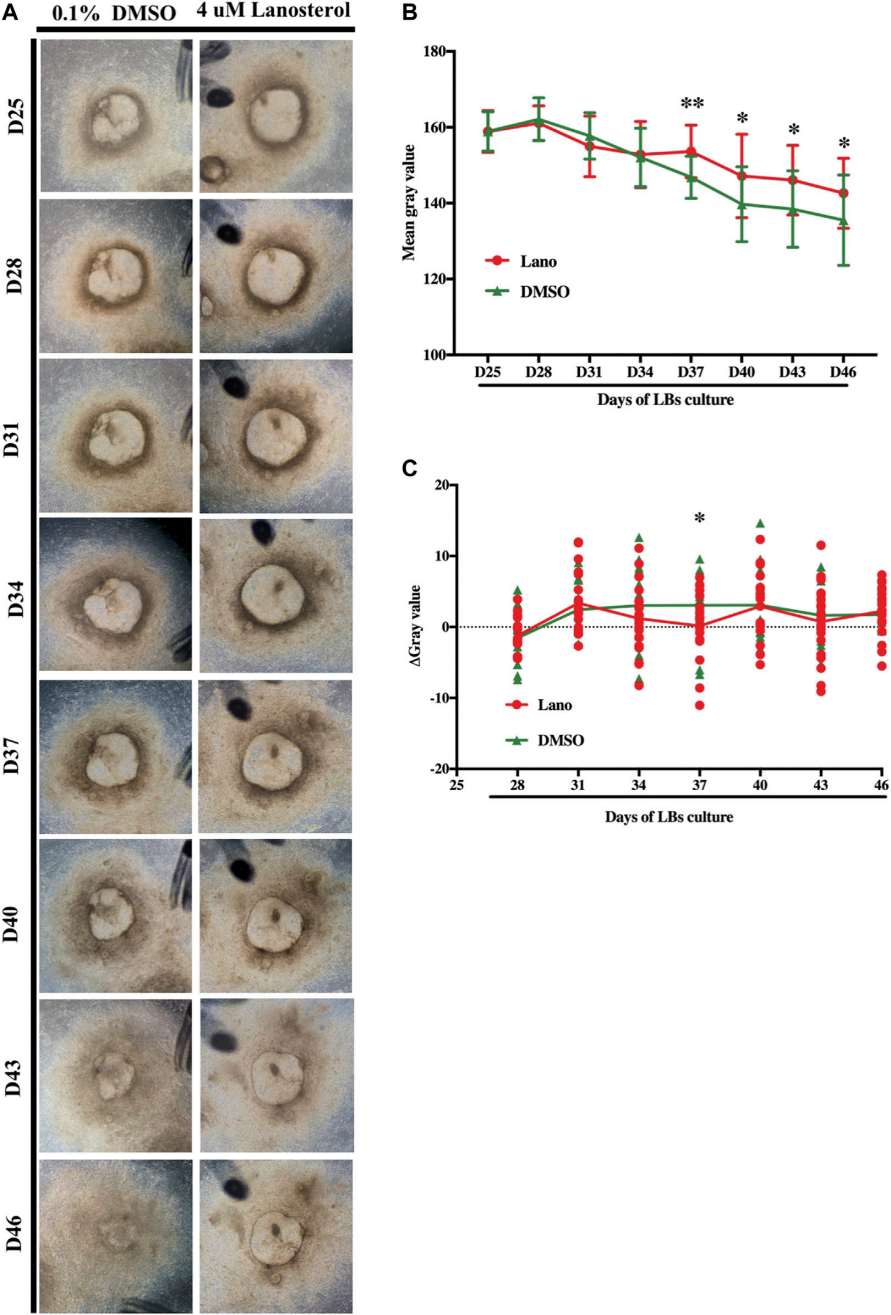
Development of lanosterol treated LBs. **(A)** Representative pictures of control and lanosterol treated LBs from D25 to D46. **(B)** The opacity degrees of LBs were evaluated by mean gray value using the ImageJ software. **(C)** The decrease rate of mean gray value of LBs was represented using ΔGray value (ΔGray value = (gray value D(n) − gray value D (n + 3))/3). At least 12 LBs in each group from three independent experiments were examined. Bars represent mean ± SD. *p < .05, **p < .01. Scale bar:1 mm. DMSO, dimethyl sulfoxide.

To further investigate the difference in clouding process between the LBs in the control group and those in the lanosterol-treated group, the LBs’ degrees of opacity were measured every 3 days between D25 and D46, using representative pictures (Figure 2A). The results showed that the LBs in both groups slowly became cloudy, and lanosterol treatment alleviated this process after D34 (Figure 2B). The ΔGray value also showed that the opacity decline rate of the lanosterol-treated LBs from D31 to D40 was lower than that of the control LBs (Figure 2C), demonstrating that before D40, the LBs’ opacification could be slowed down with drug treatment. After D40, both the lanosterol-treated and control LBs became opaque at the same speed.

### Determination of the soluble and insoluble protein concentrations of the control and lanosterol-treated lentoid bodies

**FIGURE 3:**
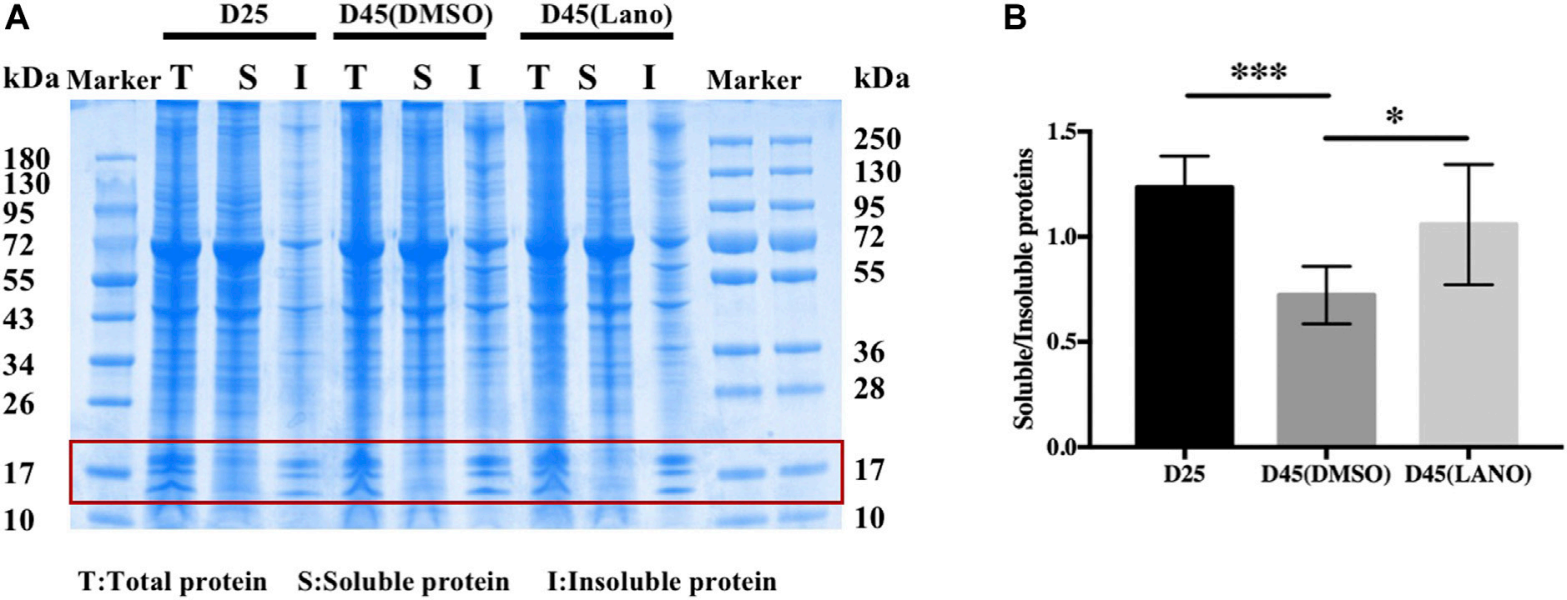
Soluble and insoluble protein concentration determination of control and lanosterol treated LBs. **(A)** Concentration of soluble and insoluble proteins was detemined by SDS-PAGE gel electrophoresis combination with coomassie blue staining. **(B)** Quantitative analysis of bands around 20kd region (red box) showed that the increase of insoluble protein was less in the lanosterol treated LBs than the control LBs. Bars represent mean ± SD (*n* = 4). *p < .05, ***p < .001. T, total protein; S, soluble protein; I, insoluble protein. DMSO, dimethyl sulfoxide. Lano, lanosterol.

Increased insoluble protein caused by protein aggregation is the basic pathological mechanism of lens opacification and cataractogenesis. (Moreau and King, 2012). We found in our previous studies that there are more cells containing protein aggregates in clouding LBs. (Qin et al., 2019). Therefore, we wanted to determine if lanosterol’s prevention of the LBs’ opacification was related to the decreased insoluble protein. We thus performed quantitative analysis via SDS-PAGE gel electrophoresis combined with Coomassie blue staining to determine the soluble and insoluble protein concentrations. The study results showed that there was more soluble protein in the transparent LBs than in the cloudy LBs, and that there was less insoluble protein increase in the cloudy lanosterol-treated LBs than in the cloudy control LBs (Figure 3B).

### Protein aggregation in the control and lanosterol-treated lentoid bodies

**FIGURE 4:**
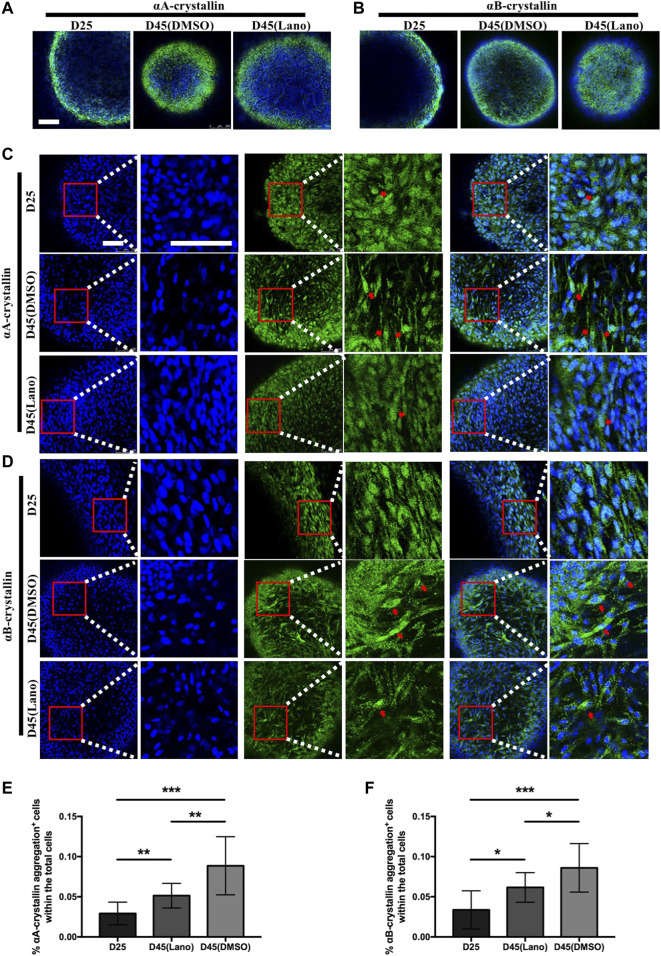
Protein aggregation in control and lanosterol treated LBs. **(A,B)** Immunofluorescence examination showed the expression of αA‐crystallin and αB‐crystallin in LBs on D25 and D45. **(C,D)** Cells containing αA‐crystallin or αB‐crystallin protein aggregations (red arrows) were highly abundant in control cloudy LBs on D45 compared with lanosterol treated LBs on D45. **(E,F)** Quantification data showed that more cells containing protein aggregations were observed in control cloudy LBs on D45 compared with control LBs on D25 and lanosterol treated LBs on D45. Bars represent mean ± SD. *p < .05, **p < .01, ***p < .001. Bar: 250um **(A,B)**, 100um **(C,D)**. DMSO, dimethyl sulfoxide. Lano, lanosterol.

**FIGURE 5:**
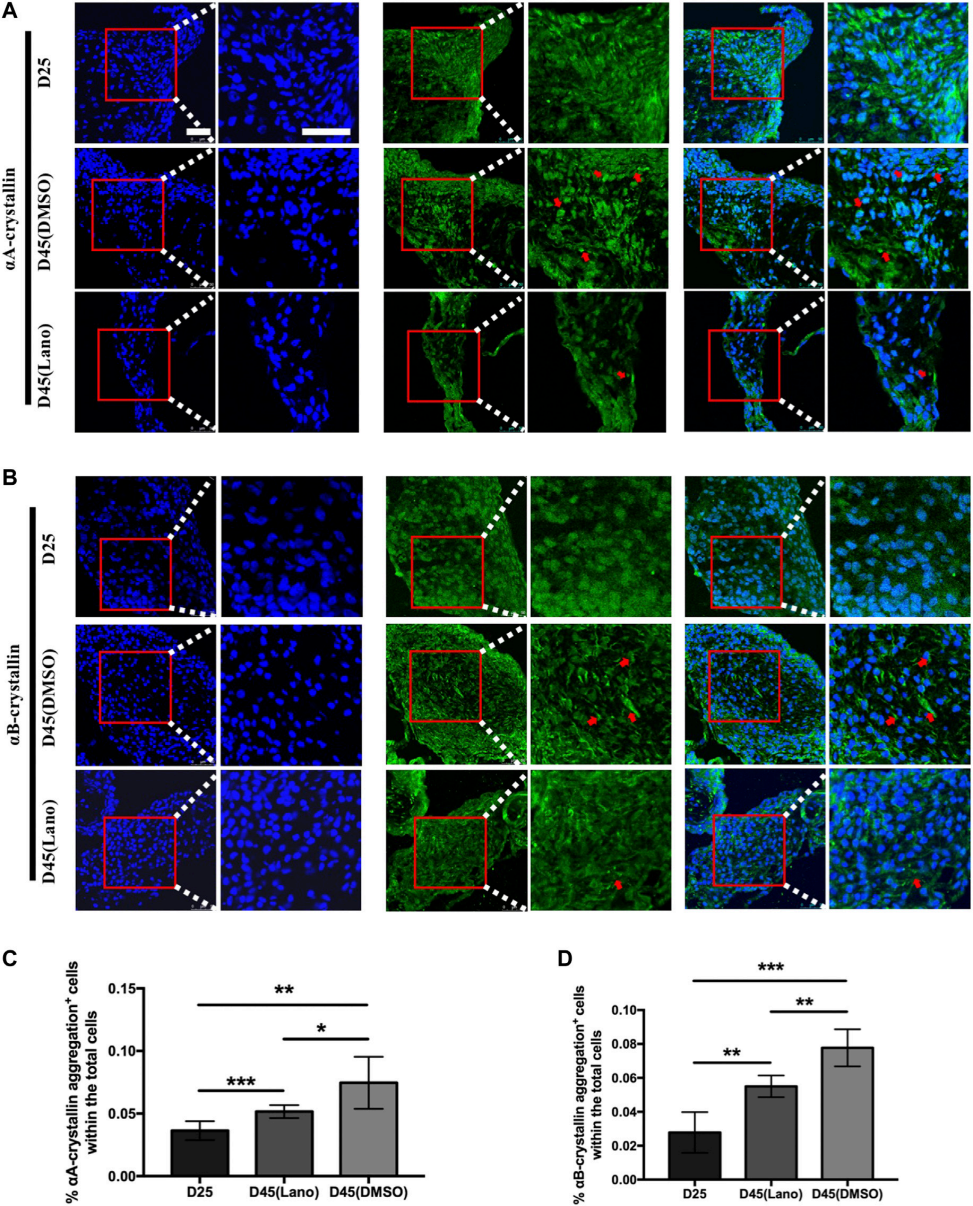
Protein aggregation in control and lanosterol treated LBs in LB slices staining. **(A,B)** Similar with Figure 4, cells containing αA‐crystallin or αB‐crystallin protein aggregation (red arrows) were more abundant in control cloudy LBs on D45 compared with lanosterol treated LBs on D45, and ware nearly absent in all LBs on D25. **(C,D)** Quantification data showed that more cells containing protein aggregations were observed in control control LBs on D45 compared with control LBs on D25 and lanosterol treated LBs on D45. Bars represent mean ± SD. *p < .05, **p < .01, ***p < .001. Bar:50uM. DMSO, dimethyl sulfoxide. Lano, lanosterol.

The soluble and insoluble protein concentration determination in the present study showed that lanosterol treatment could increase LBs’ protein solubility. As crystallin proteins are the main structural proteins present in human lenses, we used immunofluorescence staining to examine the expression patterns of αA-crystallin and αB-crystallin in the lanosterol-treated and control LBs. The results that we obtained showed that both the lanosterol-treated and control LBs expressed αA-crystallin and αB-crystallin (Figures 4A,B). In addition, cells containing protein aggregations were highly abundant in the cloudy control LBs at D45 whereas there were fewer protein aggregations in the lanosterol-treated LBs at D45 and protein aggregations were nearly absent in all the LBs at D25 (Figures 4C,D). The quantification data also showed that there were more cells containing protein aggregations in the cloudy control LBs than in the lanosterol-treated LBs at D45 (Figures 4E,F). These results suggest that protein aggregations may be the main causes of LBs’ opacification, and lanosterol may alleviate the opacification of LBs by reducing the aggregation of crystallin proteins. The staining of the LB slices also confirmed the aforementioned results (Figure 5).

## Discussion

**FIGURE 6:**
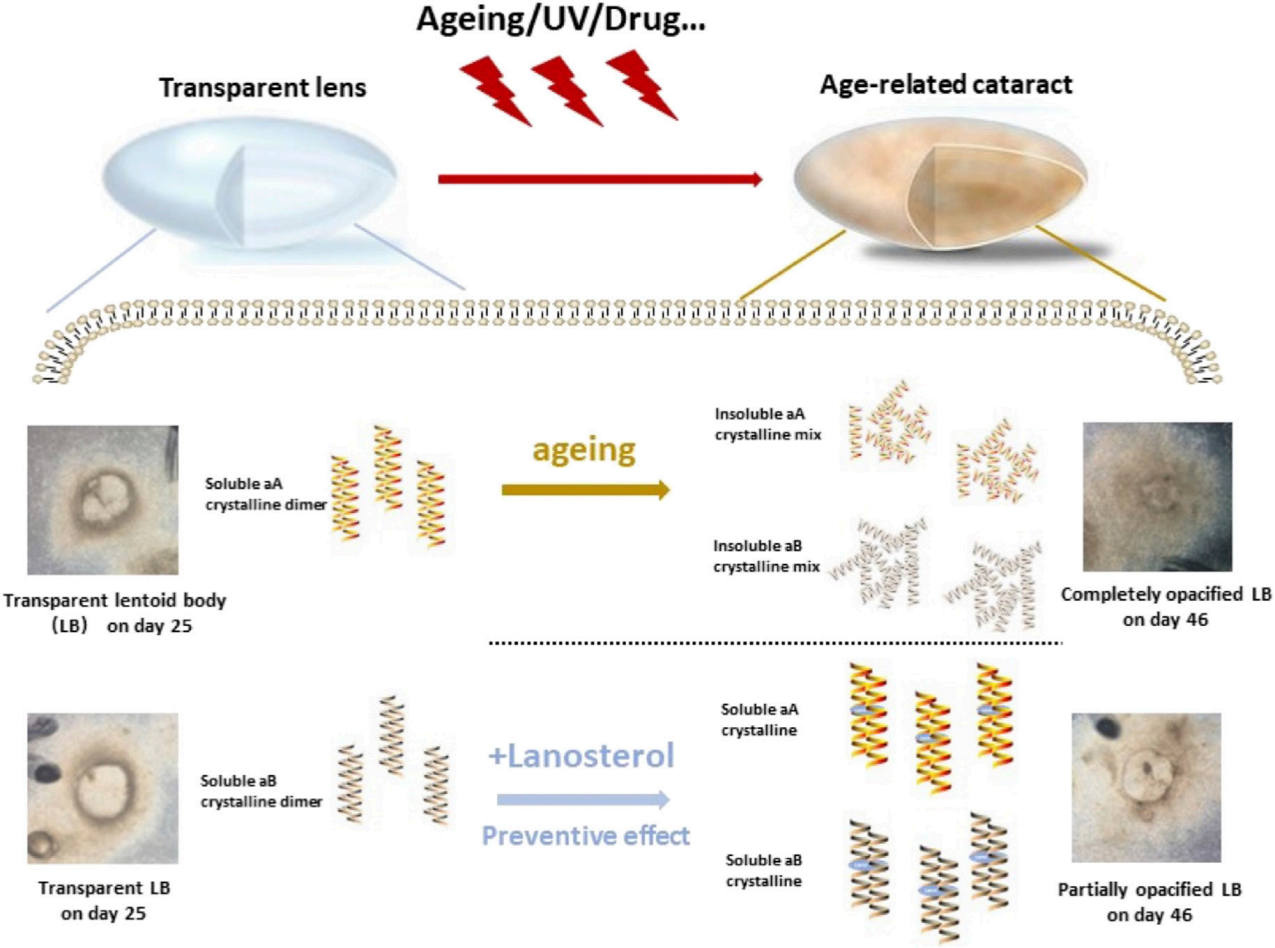
Graph abstract. The present study demonstrated that Mature LBs became cloudy with extended culture time while the opacity of LBs could be partially prevented by lanosterol treatment through maintaining the soluble of crystallin dimer.

The HiPSCs derived from LBs gradually became opaque with extended culture time and partially mimicked the lens aging process *in vitro*. (Qin et al., 2019). In the present study, we further observed that the LBs’ aging process could be postponed with lanosterol treatment, which was consistent with the lower amount of protein aggregation, indicating the potential application of the LB opacification model in drug screening for ARCs (Figure 6).

The present study provided the first evidence of the potential application of the HiPSC-derived ARC model in drug screening. HiPSCs combined with *in vitro* directed differentiation technology have been realized in various human tissues and organs, and a variety of disease and drug-screening models have been constructed on the basis of such. HiPSC-derived drug-screening models have been applied to many diseases, with promising results. Cayo et al. used hepatocytes from familial hypercholesterolemia iPSCs in a drug test to reveal that cardiac glycosides have the potential to be used for the treatment of hypercholesterolemia. (Cayo et al., 2017). Doulatov et al. also used hematopoietic progenitor cells from Diamond-Blackfan anemia iPSCs for drug discovery. (Doulatov et al., 2017). The Duchenne muscular dystrophy iPSC-derived myoblast drug-screening model and other iPSC-derived disease and drug-screening models were also applied in the locomotor system, and two promising effective small molecule drugs were found. (Sun et al., 2020). Such iPSC-derived drug-screening models could be used as continuous sources of human tissues and cells in large-scale drug-screening experiments. A total of 1,524 small-molecule compounds could be screened with high efficiency in a 96-well plate using the Duchenne muscular dystrophy iPSC-derived myoblast drug-screening model, and Cayo et al. even tested 2,320 small molecules using hepatocytes from familial hypercholesterolemia iPSCs. (Cayo et al., 2017; Sun et al., 2020).

The cloudy LB model was constructed in our previous study (Qin et al., 2019), and its potential application as an anticataract drug-screening platform was first tested with lanosterol in the present study. Our study provided the first three-dimensional human-derived ARC drug-screening platform. Similar to the aforementioned model, this platform can perform large-scale drug-screening and high-throughput experiments in a short period of time. In addition, as our model is patient-specific, it can also realize individualized study of cataract pathogenesis and precise drug therapy. Our LB model is different from most of the aforementioned models in that it is not only a simple cell model but also an organoid model with a three-dimensional structure and lenses’ optical properties. The existence of intact and undamaged lens capsule and epithelial cells (Fu et al., 2017; Qin et al., 2019) enables the LB model to better simulate the effects of drugs on the human lens. The lens capsule is a transparent and uninterrupted basement membrane secreted by the lens epithelial cells that completely encases the ocular lens, which contains collagen IV and laminin, among others. (Danysh and Duncan, 2009). It is multifunctional, provides structural support and cell signaling, and protects the lens from infectious agents while allowing small molecules such as glucose, salts, and waste products to freely pass through the capsule, thus maintaining the stability and transparency of the lens. (Danysh et al., 2010). Similarly, there are certain barriers to foreign drugs. After passing through the corneal or scleral barriers, a drug molecule used on the ocular surface will generally be distributed in the aqueous and iris/ciliary body. Before entering and taking effect in the lens cortex, drug molecules from the aqueous have to pass through the lens capsule. (Thrimawithana et al., 2018). Therefore, the presence of the lens epithelium and capsule in the LB drug-screening model can allow a more accurate simulation of the influence of drug penetration on the drug effect.

The ocular lens, an avascular and transparent tissue, contains high concentrations of crystallin proteins in the lens fibers, which contribute to lens transparency and refractive properties. The cumulative effects of lifetime exposure to radiation, oxidation, and post-translational modifications cause lens aging, resulting in protein aggregation and lens opacification, which affect light transmission, image formation, and, eventually, cataracts. (Bron et al., 2000; Moreau and King, 2012). Our previous study found that the extended-culture-time LBs became cloudy with increased protein aggregates, suggesting a similarity with human lens aging, which also involves the formation of high-molecular-weight aggregates, (Bron et al., 2000), indicating that a cloudy LB system can provide a molecular biology basis for efficacy evaluations of anticataract drugs. In this study, we found that lanosterol treatment could partially postpone the opacification of extended-culture-time LBs. However, we found that lanosterol could not reverse the opacity of LBs, it had no apparent therapeutic effect but had a preventive effect. The prevention effect of lanosterol is achieved by preventing crystallin aggregation, which is consistent with the mechanism of drug action reported in a previous study. (Zhao et al., 2015). This indicates that the mechanism of action of the selected test drug on our drug-screening platform (reducing protein aggregation) is consistent with the previously reported one. Protein aggregation is the cause of LBs’ opacification, which is also consistent with the pathogenesis of cataract. Reducing protein aggregation or even depolymerizing protein aggregates is an important target for cataract drug therapy. Our study demonstrated that an LB model could be used for anticataract drug screening through this pathological mechanism. There are many other potential targets for cataract drug therapy, such as antioxidants, which have been used as targets for anticataract drugs in many studies. (Rooban et al., 2009; Vibin et al., 2010; Jiang et al., 2019). Further studies on cataract pathogenesis and drug screening can be carried out on the basis of other cataract risk factors, such as oxidative/antioxidant ionic imbalance, and the LB model may provide an alternative platform for these studies.

The drug-screening model for ARC can involve large-scale culture as organs in a dish for mass drug screening. In addition, unlike ARC in animals with natural aging, whose development may take several years, the experimental period of our drug-screening model is shorter (about 50 days), which greatly reduces the drug-screening study period.

The proposed drug-screening model, however, has some limitations. First, LB drug-screening platforms need to be standardized. The LBs developed in this study differed in size, shape, and transparency. This may affect the accuracy of the drug-screening results. We are currently trying to overcome this shortcoming through repeated experiments, but in the follow-up study, we should further optimize the LBs’ culture method to make the LBs’ morphologies the same as much as possible. For example, we can seed a single iPSC in a 96-well plate to achieve LBs’ standardized culture and facilitate high-throughput drug screening. Second, whether the different iPSC cell lines derived from LBs have different responses to the same drug can be tested in future studies to achieve accurate screening of personalized anticataract drugs.

In summary, a new, promising HiPSC-derived ARC drug-screening model was first reported in this study. The model’s opacification can be delayed by effective drug treatment, which was achieved by reducing protein aggregation. This model is expected to be used as a new model for drug-screening and efficacy verification, and to further realize high-throughput and precise cataract drug screening.

## Data Availability

The original contributions presented in the study are included in the article/supplementary material, further inquiries can be directed to the corresponding authors.
